# Production of intracellular selenium-enriched polysaccharides from thin stillage by *Cordyceps sinensis* and its bioactivities

**DOI:** 10.3402/fnr.v60.30153

**Published:** 2016-02-01

**Authors:** Shengli Yang, Hui Zhang

**Affiliations:** 1 The College of Pharmaceutical Science, Zhejiang University of Technology, Hangzhou, People's Republic of China; 2 Zhejiang Institute of Quality Inspection Science, Hangzhou, People's Republic of China

**Keywords:** thin stillage, intracellular selenium-enriched polysaccharides, antioxidant activities, fermentation, response surface methodology, *Cordyceps sinensis*

## Abstract

**Background:**

Thin stillage was used as the substrate to produce intracellular selenium-enriched polysaccharides (ISPS) from *Cordyceps sinensis* to increase the value of agricultural coproducts.

**Methods:**

Fermentation parameters were optimized using response surface methodology (RSM) to improve the production of ISPS. Then, the effects of ISPS on the antioxidant activities *in vitro*, as well as the glycosylated serum protein concentration, malondialdehyde level, and total antioxidant capacity of streptozotocin-induced diabetic rats were studied.

**Results:**

The optimized conditions were as follows: sodium selenite concentration, 33.78 µg/L; incubation time, 8.24 days; and incubation temperature, 26.69°C. A maximum yield of 197.35 mg/g ISPS was obtained from the validation experiments, which was quite close to the predicted maximum yield of 198.6839 mg/g. FT-IR spectra indicated that ISPS has been successfully selenylation modified with similar structure to polysaccharide of intracellular polysaccharides. The *in vitro* scavenging effects of 1.0 mg/mL ISPS on hydroxyl, superoxide, and 1,1-diphenyl-2-picrylhydrazyl radicals were 74.62±4.05, 71.45±3.63, and 79.48±4.75%, respectively. The reducing power of ISPS was 0.45±0.01 (absorbance at 700 nm). Fasting blood glucose and glycosylated serum protein of group C (rats with diabetes that received drinking water with ISPS) were significantly lower than those of group B (rats with diabetes) (*P*<0.01) after treatment was administered for 2 and 4 weeks. Serum malonaldehyde content of group C was significantly lower than that of group B at 4 weeks (*P*<0.01). At 4 weeks, malonaldehyde contents in heart, liver, and kidney tissues of group C were significantly lower than those of group B; however, malonaldehyde content in pancreas tissue of group C was not significantly different. Total antioxidant capacities in liver, pancreas and kidney tissues of group C were significantly higher than those of group B, but total antioxidant capacity in heart tissue was not significantly different. Serum total antioxidant capacity was also increased compared with that of group B.

**Conclusion:**

The result of these experiments indicated that RSM is a promising method for the optimization of ISPS production, and the ISPS of *C. sinensis* can reduce blood glucose level and improve antioxidant capacity of rats with diabetes induced by streptozotocin.

*Cordyceps*, a genus of Ascomycetes (sac fungus), include approximately 400 identified species and various species that remain undescribed. All *Cordyceps* species are entomopathogenic endoparasitoid fungi because they develop mainly in insects and other arthropods (1), while a few of them are parasitic on other fungi (2). Cultivated *Cordyceps* mycelia exhibit chemical composition and clinical efficacy similar to those of natural *Cordyceps*, but with reduced toxicity (3). *C. sinensis* is a prominent tonic of concentrated precious herbs beneficial for the lung, liver, heart, and kidney of humans, which has been used to treat several diseases such as arrhythmia, palpitation, asthma, and chronic nephritis, among others (4). Similar to medicinal mushrooms, *C. sinensis* is used to produce mycelium and physiologically active substances by submerged fermentation. As an important active substances produced by *C. sinensis*, polysaccharides have potential antioxidant (5, 6), antitumor (7), antiviral (8), and immunomodulating properties (9–11).

Selenium is one of the indispensable elements for human and animals, which can be found in various animal organs such as liver, kidney, and pancreas. Selenium plays an important role in metabolism, reproduction, and immunity. Several recent studies have focused on the biotransformation of selenite, in which microorganisms were fermented in the presence of varied amounts of selenium to produce selenium-enriched products (12). The selenium-enriched yeast is the most widely investigated nutritional supplement that contains selenium. During the growth of *Saccharomyces cere*v*isiae* yeast, inorganic selenium, a potentially toxic species with low bioavailability, is converted to safer organic selenium species (13). Organic selenium enhances the selenium level in the body. Moreover, it increases the activity of glutathione peroxidase, which protects cell membrane, prevents the formation of free radicals that cause cellular damage, and improves antioxidant status for better immune function. Consequently, organic selenium can prevent and treat diseases.

Thin stillage is a low valued waste, except that small part of it is used as feed. However, thin stillage contains various unfermented components of corn and yeast cells. These compounds are ideal nutrients for microorganisms. Thin stillage may generate microbial mycelia and valuable products (14, 15). In this study, thin stillage was used as medium of *C. sinensis* for the production of intracellular selenium-enriched polysaccharides (ISPS) by submerged fermentation to increase the additional value of alcohol production. Further optimization of the culture conditions is essential to obtain a higher production yield. Thus, we attempted to optimize fermentation parameters using response surface methodology (RSM) to improve the production of ISPS. As an efficient strategy for determining the optimal conditions for a multi-variable system with a rational number of experiments, RSM has already been successfully applied in the optimization of numerous fermentation processes (16–19). In addition, sodium selenite (SSe) was added into thin stillage to produce ISPS from *C. sinensis* to improve the nutritional value of polysaccharide. Finally, the antioxidant activities of the ISPS important in food and pharmaceutical industries were evaluated.

## Material and methods

### Microorganisms

The *C. sinensis* strain used throughout this study was preserved in our laboratory.

### Materials

A total of 32 healthy male Sprague-Dawley (SD) rats were purchased from the Laboratory Animal Center of Zhejiang University (Institutional Animal Welfare and Ethics Committee in Zhejiang University in China). This study was approved by the Animal Ethics Committee in Zhejiang University. Streptozotocin was purchased from Sigma-Aldrich, St. Louis, USA. Protamine zinc insulin was purchased from Shanghai No. 1 Biochemical & Pharmaceutical Co., Ltd. (Shanghai City, China). Detection kits of glycosylated serum protein, malonaldehyde, and total antioxidant capacity were purchased from Shanghai Tong Wei Industrial Co., Ltd. (Shanghai City, China).

### Seed culture and inoculum preparation

The fungi were cultivated on potato dextrose (PD) agar containing 2.0% agar and incubated at 25°C for 7 days, stored at 4°C, and subcultured every 3 months.

### Submerge fermentation

A 0.5-cm^2^ mycelial block of *C. sinensis* was transferred to shake flasks (250 mL) containing 50 mL of thin stillage. The flasks were shaken at 180 rpm at 25°C for 24 h. A 10% (v/w) inoculum was transferred to 50 mL of fermentation medium in a 250 mL flask. The compositions of the fermentation medium were 50 mL thin stillage with different amounts of SSe. The pH was adjusted to 7.0 with 0.1 M of NaOH before autoclaving the media at 121°C for 15 min. The temperature and time were set to the levels of experimental design. All treatments were conducted three times.

### ISPS preparation and measurement

After fermentation, liquid fungal biomass was harvested using a piece of gauze with a pore size of 100 µm. The biomass was washed three times with distilled water, and dried to a constant weight at 60°C for 24 h. The dry mycelia from *C. sinensis* were washed and degreased with ether at 60°C for 18 h in a water bath. The residues were collected by centrifugation (4,000×*g* for 15 min) and dried at room temperature. Then, they were extracted three times with distilled water for 2 h in a boiling water bath, and then centrifuged at 4,000×*g* for 15 min. The aqueous extracts were pooled and concentrated to one-fifth of its total volume. The concentrated liquors were dialyzed against water, and the non-dialyzable solutions were reconcentrated. The supernatant was mixed with three volumes of 95% ethanol (v/v), stirred vigorously, and left overnight at 4°C. The precipitated polysaccharides were centrifuged at 8,000×*g* for 20 min, and the supernatant was discarded. The polysaccharide precipitates were washed three times with 70% ethanol and lyophilized to a constant weight in vacuo. The weight of ISPS was estimated. The product yield was measured at (w/w) % of polysaccharides per unit mass of lyophilized mycelium.

### Design of experiments

A central composite design at three levels was performed to determine the effects of the dispersion condition variables on the response. The SSe concentration, incubation time, and incubation temperature were selected as independent variables. The range of the values and coded levels of the variables are listed in Table 1. A polynomial equation was used to predict the response as a function of independent variables and their interactions. In this study, the number of independent variables is three. Therefore, the response for the quadratic polynomials is as follows:

$$\text{Y}=\beta_{\text{0}}+\beta_{\text{1}}{\text{X}}_{\text{1}}+\beta_{\text{2}}{\text{X}}_{\text{2}}+\beta_{\text{3}}{\text{X}}_{\text{3}}+\beta_{\text{12}}\;{\text{X}}_{\text{1}}\;{\text{X}}_{\text{2}}+\beta_{13}X_{1}\;{\text{X}}_{\text{3}}+\beta_{\text{23}}{\text{X}}_{\text{2}}{\text{X}}_{\text{3}}+\beta_{\text{11}}{\text{X}}_{\text{1}}^{\text{2}}+\beta_{\text{22}}+{\text{X}}_{\text{2}}^{\text{2}}+\beta_{\text{33}}{\text{X}}_{\text{3}}^{\text{2}}$$

**Table 1:** Independent variables and their coded levels and values

		Coded level		
		
Variable	Symbol	−1	0	1
Sodium selenite concentration (µg/mL)	X_1_	10	25	50
Incubation time (day)	X_2_	6	7	8
Incubation temperature (°C)	X_3_	20	25	30

where Y is the predicted response, X_1_ is the SSe concentration, X_2_ is the incubation time, X_3_ is the incubation temperature, β_0_ is the intercept coefficient, β_j_ denotes the linear terms, β_ij_ denotes the interaction terms, and β_jj_ denotes the square terms.

### Statistical analysis

Data were processed using analysis of variance (ANOVA) to obtain the interaction parameters between the process variables and response. The fitting quality of polynomial model was expressed by the coefficient of determination *R*^2^ and its statistical significance was verified by the F-test.

### Infrared spectra analysis

The infrared (IR) spectra were recorded using the KBr-disc method with a Fourier transform infrared (FTIR) spectrometer (Tensor27 Fourier transform infrared spectrometer; Bruker, Germany) in the range of 400–4,000 cm^−1^.

### Hydroxyl radical scavenging assay

Hydroxyl radical scavenging activity was assayed by the method of Winterbourn and Sutton (20). The solutions included 1 mL 0.945 mM EDTA–Fe (II), 1 mL 0.15 M phosphate buffer (pH 7.4), 1 mL 3% (v/v) H_2_O_2_, 1 mL 40 µg/mL safranin, 0.5 mL ISPS (0.1–1.0 mg/mL), 0.5 mL intracellular polysaccharides (IPS) (0.1–1.0 mg/mL), and 0.5 mL SSe (0.1–mg/mL). After incubation at 37°C for 30 min, the absorbance was measured at 560 nm using BHT (butylated hydroxytoluene) as positive control. The hydroxyl radical scavenging activity was calculated using the following formula:

1 $$\text{Scavenging rate }(\%)=\times \left(\frac{A_{0}-A_{1}}{A_{0}}\right)100\%$$

where *A*_1_ is the absorbance of ISPS, IPS, SSE, and BHT, and *A*_0_ is the absorbance of the blank.

### Superoxide radical scavenging assay

Superoxide anion radical scavenging activity was assayed by the method of Stewart and Beewley (21). The reaction solutions (3 mL) included 75 µM nitro blue tetrazolium, 13 mM methionine, 50 mM phosphate buffer (pH 7.8), 10 mM riboflavin, 100 mM EDTA, the ISPS (0.1–1.0 mg/mL), the IPS (0.1–1.0 mg/mL) and the SSe (0.1–1.0 mg/mL). The mixed solution was incubated at 25°C for 30 min in a water bath, and the absorbance of the samples was measured by reading the absorbance at 560 nm, using BHT as positive control. The whole reaction was loaded in a box lined with aluminum foil. The scavenging rate was evaluated using the following formula:

2 $$\text{Scavenging rate }(\%)=\times \left(\frac{A_{0}-A}{A_{0}}\right)100\%$$

where *A*_1_ is the absorbance of ISPS, IPS SSe, and BHT, and *A*_0_ is the absorbance of the blank.

### 1,1-diphenyl-2-picrylhidrazyl (DPPH) scavenging assay

The DPPH scavenging activity of the samples was evaluated as described by the method of Shimada (22). The mixture contained 0.1 µM DPPH, 2 mL 95% ethanol, and 2 mL different concentrations (0.1–1.0 mg/mL) of ISPS, IPS, and SSe. The reaction mixture was incubated at 25°C for 15 min, and the absorbance of samples was followed by monitoring at 517 nm using a UV-spectrophotometer, using BHT as positive control. The antioxidant activity of ISPS was calculated according to the following equation:

3 $$\text{Scavenging rate }(\%)=\times \left(\frac{1-A}{A_{0}}\right)100\%$$

where *A*_0_ is the absorbance of the DPPH solution, and *A* is the absorbance of ISPS, IPS, SSe, and BHT.

### Determination of reducing power

The reducing power of the samples was assayed by the method of Oyaizu (23). The reaction solutions contained 2.5 mL potassium ferricyanide (1%, w/v), 2.5 mL phosphate buffer (pH 6.6, 0.2 M), the ISPS (0.1–1.0 mg/mL), the IPS (0.1–1.0 mg/mL), and the SSe (0.1–1.0 mg/mL). After reacting at 50°C for 20 min in a water bath, 2.5 mL trichloroacetic acid (10%, w/v) was added to the mixture to terminate the reaction. Then the mixture was centrifuged at 12,000×g for 10 min. The 2.5 mL supernatant was collected and mixed with 0.5 mL FeCl_3_ (0.1%, w/v) and 2.5 mL deionized water. The mixed solution was incubated at room temperature for 15 min, and the absorbance of the samples was assayed at 700 nm, using BHT as positive control.

### Establishment of a rat model

Among the 32 SD rats, 26 were subjected to fasting but were provided drinking water for 12 h. The rats were intraperitoneally injected with 65 mg/kg streptozotocin (dissolved in 0.1 mmol/L buffer solution of citric acid–sodium citrate before use to obtain a final concentration of 10 mg/ml, pH 4.4) to establish the diabetic rat model. Citric acid–sodium citrate buffer solution (6.5 ml/kg) was also intraperitoneally injected into the six remaining rats of the same age. At 72 h after streptozotocin was injected, blood glucose level of >16 was obtained. Urine glucose level of 7–8 mmol/L was also determined. For 2 consecutive days, polydipsia, polyphagia, urorrhagia, and body weight loss were found.

### Experimental groups and disposal

Normal control group (group A) consisted of six rats of the same age which were injected intraperitoneally with buffer solution containing no streptozotocin. The 24 rats used for the diabetic rat model were then randomly divided into three groups (*n*=8 in each group) according to blood sugar and body weight: 1) control group, rats with diabetes (group B), 2) rats with diabetes that received drinking water with ISPS (20 mg/L; group C), and 3) insulin group, rats with blood glucose level controlled at 4–8 mmol/L and urine glucose kept negative by subcutaneously injecting protamine zinc insulin (daily injection of 10–20 U/kg; group D). Before treatment was administered, fasting blood glucose levels in each group were determined. Two weeks after the treatment, fasting blood glucose levels of rats in groups A, B, and C, and the glycosylated serum protein and serum malonaldehyde contents of the rats in all four groups were determined. Four weeks after the treatment, fasting blood glucose levels of the rats in groups A, B, and C were determined. In the same period, glycosylated serum protein content, serum malonaldehyde content, and total antioxidant capacity of the rats in all the groups and malonaldehyde content and total antioxidant capacity in tissue homogenates of the heart, the liver, the pancreas, and the kidney were also determined.

### Determination of observation indexes

A drop of blood was obtained by bobtail method after anesthesia. Random blood sugar and fasting blood glucose levels were determined using Accu-Chek (German) blood glucose meter and blood glucose test strips. A small amount of urine was collected from metabolic cages, and urine glucose level was then measured using urine glucose test strips. Glycosylated serum protein content, malonaldehyde content, and total antioxidant capacity were then determined by blood sampling via the canthus vein. Tissue homogenate was prepared after the rats were sacrificed by cervical dislocation, and then the glycosylated serum protein content, malonaldehyde content, and total antioxidant capacity were determined in strict accordance with the manufacturers’ instructions.

## Results

### Development of a regression model

Response surface optimization is more advantageous than the traditional single parameter optimization because it saves space, time, and raw material. A total of 15 runs were conducted to optimize the three individual parameters in the central composite design. The experimental conditions and results of the effect of the independent variables, namely SSe concentration, incubation time, and incubation temperature, on the ISPS production according to the factorial design are shown in Table 2. The application of RSM yielded the following regression equation models, indicating empirical relationships between ISPS production and the test variables in coded units. The relationship among the variables (as coded values), SSe concentration (X_1_), incubation time (X_2_), and incubation temperature (X_3_) was fitted by second-order polynomial equations as follows:

$$\text{Y}=197.65+5.2*{\text{X}}_{1}+3.14375*{\text{X}}_{2}+1.16125*{\text{X}}_{3}-7.1725*{\text{X}}_{1}^{2}-0.8075*{\text{X}}_{1}*{\text{X}}_{2}-0.2725*{\text{X}}_{1}*{\text{X}}_{3}-8.12*{\text{X}}_{2}^{2}+0.405*{\text{X}}_{2}*{\text{X}}_{3}-8.415*{\text{X}}_{3}^{2}$$

**Table 2:** The 3×3 factorial central composite designs and response values for condition optimization of ISPS production

Trial	X_1_	X_2_	X_3_	ISPS (mg/g)
1	−1	−1	0	172.64
2	−1	0	−1	175.83
3	−1	0	1	178.55
4	−1	1	0	181.02
5	0	−1	−1	177.38
6	0	−1	1	179.04
7	0	1	−1	182.38
8	0	1	1	185.66
9	1	−1	0	185.31
10	1	0	−1	186.12
11	1	0	1	187.75
12	1	1	0	190.46
13	0	0	0	197.66
14	0	0	0	198.07
15	0	0	0	197.22

The corresponding ANOVA of each empirical model obtained along with the values of the determination coefficient (*R*^2^) and the adjusted determination coefficient (adj. *R*^2^) are presented in Table 3. Statistical analysis indicated that the proposed model was adequate and presented satisfactory values of *R*^2^. The closer the value of *R*^2^ to unity, the better the empirical model fitted the actual data. The *R*^2^ value for ISPS production was 0.9981, indicating that the regression models explained at least 99.81% of the total variation in the responses. The probability (*P*) values of all regression models were less than 0.05. These facts indicated that the quadratic models described the experimental data satisfactorily.

**Table 3:** ANOVA for the second-order polynomial equation

Source	Degree of freedom	Sum of squares	Mean squares	*F*	Prob>*F*
Model	9	912.6214	101.4024	294.9501	0.0001
Error	5	1.718975	0.343795		
Total	14	914.3404			

*R*^2^=99.81%; Adj. *R*^2^=99.47%; CV=0.31693.

Table 4 clearly shows that the ISPS production was significantly affected by the linear (*P*<0.01) and quadratic (*P*<0.01) effects of SSe concentration and incubation temperature. The linear term of incubation time had a significant effect on the ISPS production as well as the interaction term of SSe and incubation time. The effect of interaction between incubation time and incubation temperature on the ISPS production was negative (*P*<0.05). The quadratic term of incubation time did not show any significant effect on ISPS production (*P*<0.05).

**Table 4:** Regression coefficients of a full second-order polynomial model for medium optimization of ISPS production

Source	Degree of freedom	Coefficients estimated	Standard deviation	*T*	Prob>∣*T*∣
X_1_	1	5.2	0.207303	25.0841	0.0001
X_2_	1	3.14375	0.207303	15.16503	0.0001
X_3_	1	1.16125	0.207303	5.601714	0.002505
X_1_*X_1_	1	−7.1725	0.305141	−23.5055	0.0001
X_2_*X_1_	1	−0.8075	0.29317	−2.75437	0.040101
X_2_*X_2_	1	−0.2725	0.29317	−0.92949	0.395295
X_3_*X_1_	1	−8.12	0.305141	−26.6106	0.0001
X_3_*X_2_	1	0.405	0.29317	1.38145	0.225684
X_3_*X_3_	1	−8.415	0.305141	−27.5774	0.0001

### Effects of process parameters on the optimization of ISPS

**Fig. 1:**
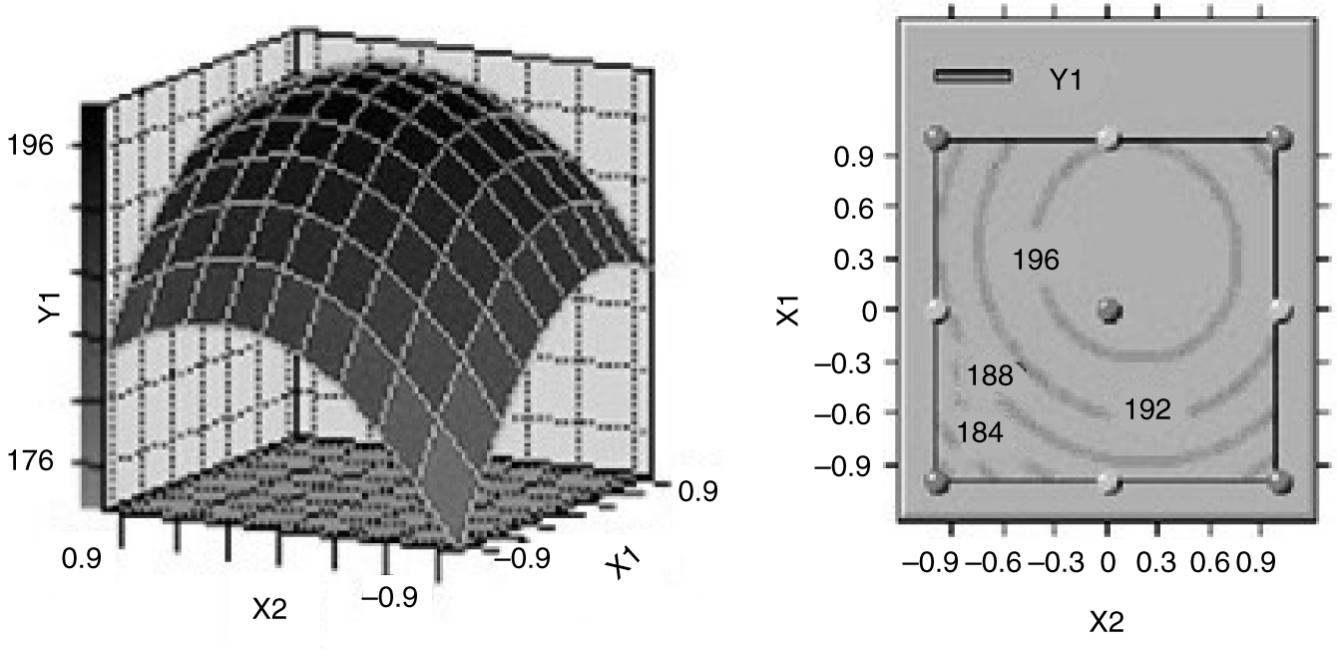
Response surface plot and contour plot of the combined effect of incubation time and sodium selenite concentration on ISPS production by *C. sinensis*.

**Fig. 2:**
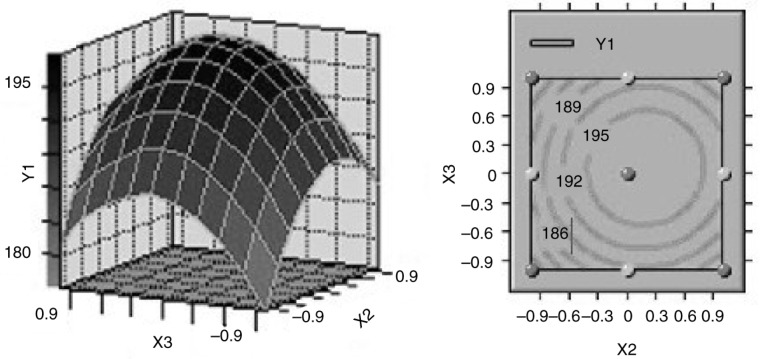
Response surface plot and contour plot of the combined effect of incubation time and incubation temperature on ISPS production by *C. sinensis*.

**Fig. 3:**
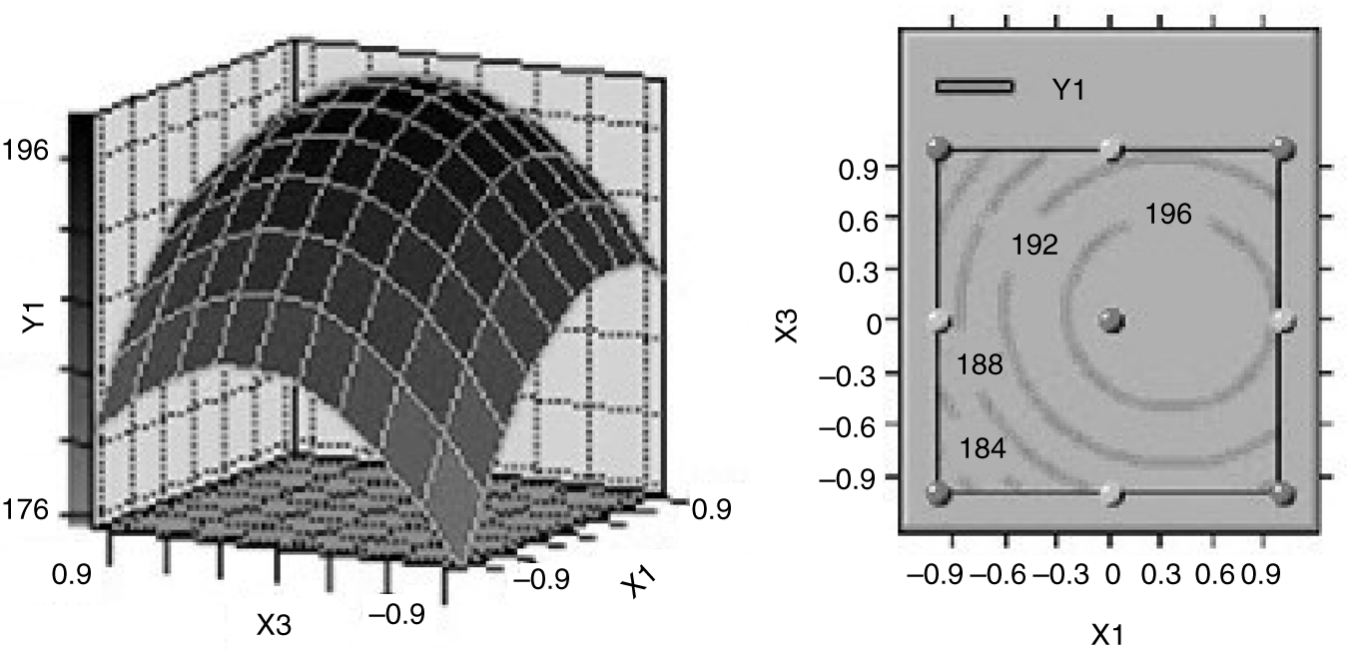
Response surface plot and contour plot of the combined effect of incubation temperature and sodium selenite concentration on ISPS production by *C. sinensis*.

Based on the variance analysis, three variables significantly affected ISPS yield. Three-dimensional (3-D) surface responses and their corresponding 2-D contours were plotted to illustrate the relationships between the responses and variables (Figs. 1–3). Each figure presents the effects of two factors, whereas the other factor was fixed at the central level. These 3-D plots and 2-D contours provide a visual interpretation of the interaction between the two factors and facilitate the determination of optimal experimental conditions. The effects of SSe concentration and incubation time on the variation of ISPS production at fixed incubation temperature are shown in Fig. 1. The minimum response of ISPS production occurred at the lowest temperature. ISPS production increased considerably with increasing SSe concentration, demonstrating that the SSe concentration significantly affected ISPS production. The responses were maximal near the middle of the SSe concentration. The response also varied at different incubation time along the axis, and the interaction between these two variables had a significant effect (*P*<0.05). The effects of incubation time and temperature on the variation of ISPS production at fixed SSe concentration is shown in Fig. 2, which indicates that the interaction between these two variables was negative (*P*<0.05). In addition, Fig. 3 shows the effect of SSe concentration and incubation temperature on the variation of ISPS production at fixed incubation time. The effects of SSe concentration and incubation temperature on the yield were curvilinear, and the interaction between them was significantly positive (*P*<0.05).

Canonical analysis, a mathematical approach used to examine the overall shape and locate the stationary point of the response surface, showed that the stationary point of the current response surface was a maximum response. At the optimal medium composition (33.78 µg/L SSe, 8.24 days incubation time, and 26.69°C incubation temperature), an ISPS production of 198.6839 mg/g was predicted. *C. sinensis* was incubated in 250-ml shake flasks containing the optimized medium to confirm the model adequacy in predicting the maximum ISPS production. A maximum ISPS production of 197.35 mg/g was obtained, which was quite close to the predicted maximum response value. Thus, the model was proven adequate.

### The infrared spectra of ISP and ISPS

**Fig. 4:**
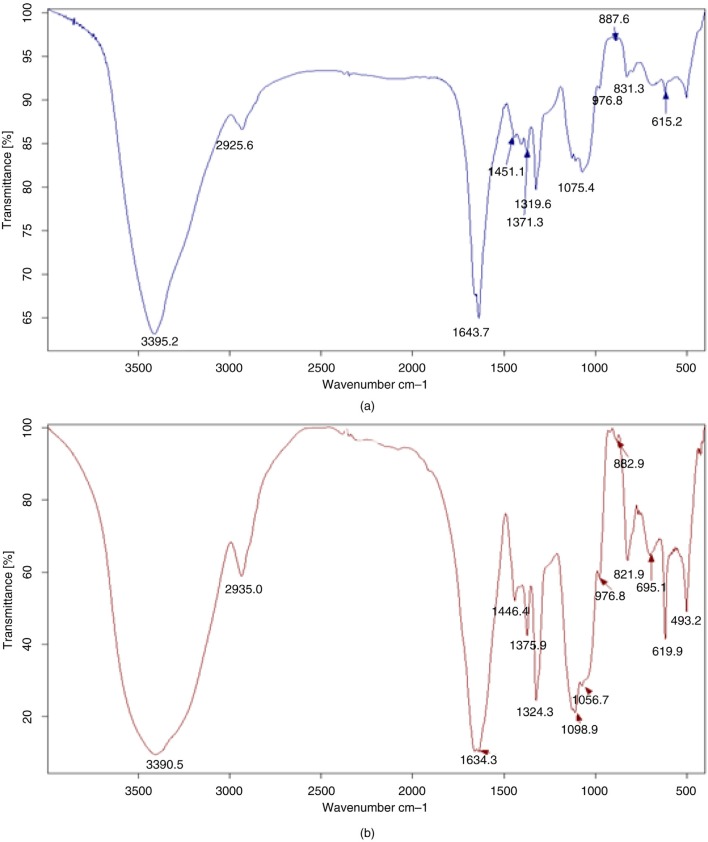
IR spectra of the IPS (a) and ISPS (b).

The infrared spectra of the purified IPS and ISPS are shown in Fig. 4. All characteristic absorptions of the polysaccharides were strong and the wide absorption bands were in the range of 3,200–3,600 cm^−1^ for O–H stretching vibrations with strong absorption peak of about 2,800–3,000 cm^−1^ for C–H stretching vibrations. The bands in the region of 2,935.0 cm^−1^ are due to C–H stretching vibration, and the bands in the region of 1,634.3 cm^−1^ are due to associated water. The absorbances of the polysaccharides in the range of 950–1,200 cm^−1^ were the instances in which the C–O–C and C–O–H link band positions were found. Compared with the spectrogram of IPS, 1 weak characteristic absorption band was at 882.9 cm^−1^, describing an asymmetrical Se=O stretching vibration of selenium ester (24), and another characteristic absorption bands was at 619.9 cm^−1^, describing an asymmetrical Se-O-C stretching vibration (25), which demonstrated that ISPS was successfully modified in selenylation.

### Antioxidant activities of ISPS in vitro

Antioxidant activities have been attributed to various reactions and mechanisms, such as radical scavenging, reductive capacity, prevention of chain initiation, and binding of transition metal ion catalysts (26). In the current experiment, the *in vitro* antioxidant capacities of IPS, ISPS, and SSe were evaluated using various biochemical methods of DPPH radical scavenging assay, superoxide anion, hydroxyl radical, and reducing power analysis.

**Fig. 5:**
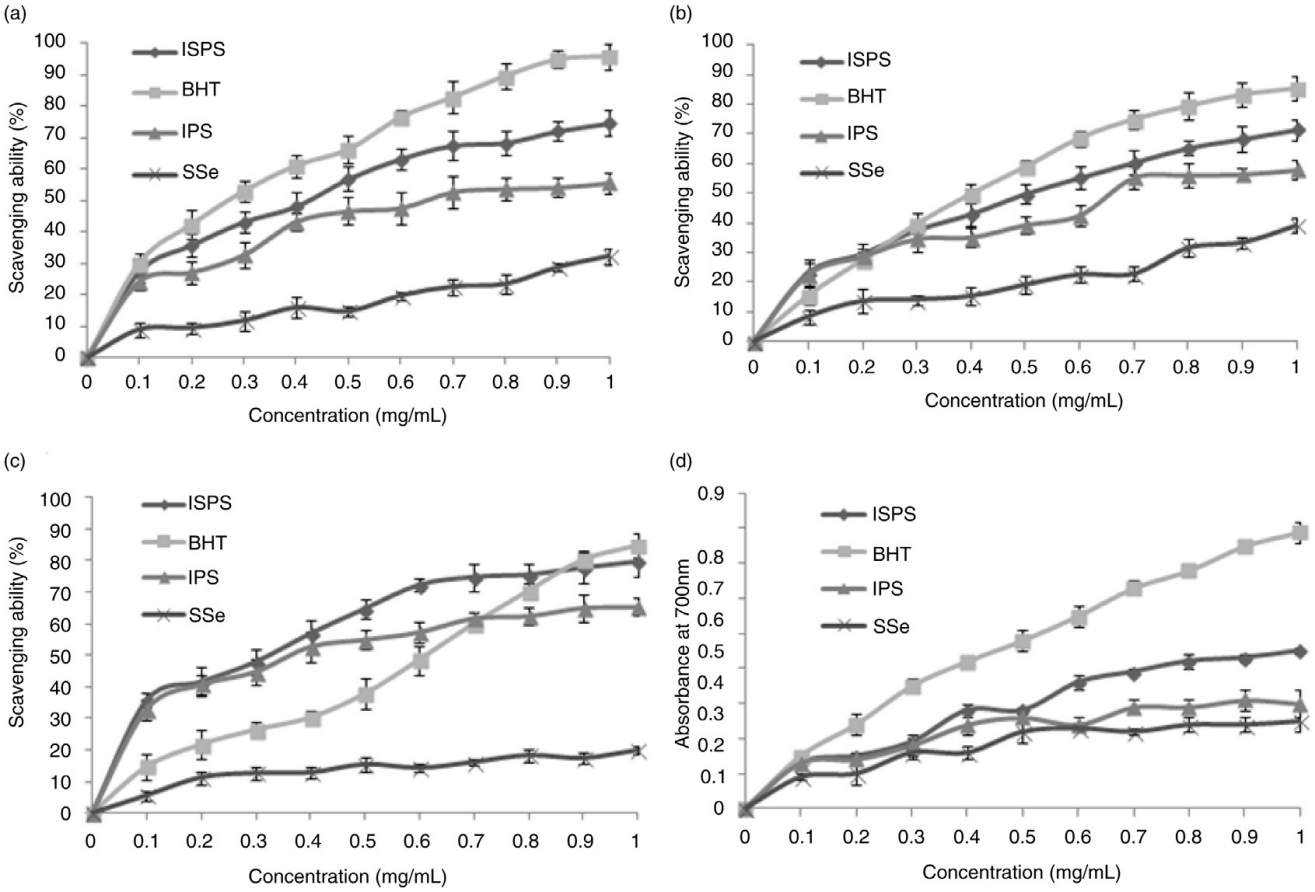
Antioxidant activities of IPS, ISPS, and SSe. (a) Scavenging of hydroxyl radical, (b) scavenging of superoxide radical, (c) Scavenging of DPPH radical and (d) reducing power.

The hydroxyl radical can easily penetrate the cell membrane and virtually damage all types of macromolecules, such as carbohydrate, protein, lipids, and DNA, resulting in necrotic cell and organic pathology (27). Thus, scavenging this radical is important because of its adverse effects (28). The hydroxyl radical has a very short *in vivo* half-life of approximately 10^−9^ sec and high reactivity (29). The results from the hydroxyl radical scavenging assay for various concentrations of IPS, ISPS, and SSe (0.1, 0.2, 0.3, 0.4, 0.5, 0.6, 0.7, 0.8, 0.9, and 1.0 mg/mL) are illustrated in Fig. 5a. Compared with the IPS and SSe, ISPS showed higher scavenging ability. The hydroxyl radical scavenging activity of ISPS was concentration-dependent and slightly lower than that of BHT in the test dosage range. The ISPS scavenging activity was 74.62±4.05% when the concentration was 1.0 mg/mL. The EC_50_ value of ISPS for the hydroxyl radical scavenging activity was 0.41±0.06 mg/L, which significantly differed from the scavenging effect of BHT (0.26±0.05 mg/L). These results proved that ISPS was a good scavenger for hydroxyl radical.

The superoxide radical is considered a relatively weak oxidant. However, it can degrade continuously and form other reactive oxygen species, such as hydrogen peroxide and hydroxyl radical, through dismutation and other types of reaction *in vivo*. The superoxide radical and its derivatives could trigger peroxidation of lipids and then induce pathological incidents, which is extremely harmful to humans (30, 31). Superoxide radicals are also very harmful to cellular components and are responsible for various diseases (32). The superoxide radical scavenging assay showed that a better antioxidant activity of IPS, ISPS, and SSe in Fig. 5b. Compared with IPS and SSe, ISPS exhibited more effective antioxidant activity. The scavenging activity increased with increasing concentration of ISPS, and BHT displayed higher scavenging ability when the ISPS concentration was lower than 0.25±0.04 mg/mL. The ISPS scavenging activity ranged from 23.08±4.47 to 71.45±3.63% when the concentration was in the range of 0.1–1.0 mg/mL. EC_50_ values of ISPS and BHT were 0.50±0.08 and 0.38±0.06 mg/mL, respectively. Although its scavenging activity was weaker than that of BHT, the ISPS are good superoxide anion scavengers at high concentrations.

The DPPH free radical is a stable-free radical widely accepted to estimate free radical scavenging activities of antioxidants (33). DPPH shows a maximum absorbance at 517 nm in ethanol. When DPPH encounters a proton-donating substance, such as an antioxidant, the radical would be scavenged, and its absorbance reduced (22). The scavenging ability of ISPS toward the DPPH radical is shown in Fig. 5c and compared with that of IPS and SSe, ISPS exhibited more effective scavenging activity. Similar to the hydroxyl and superoxide radicals, the scavenging activity toward the DPPH was related to the sample concentration. The hydroxyl radical scavenging activity of ISPS was higher than that of BHT at concentrations lower than 0.87±0.05 mg/mL. The scavenging ability of ISPS toward the DPPH radical was 35.64±2.28% at 0.1 mg/mL, which increased to 79.48±4.75% at 1.0 mg/mL in a concentration-dependent manner. The EC_50_ values of the ISPS and BHT were similar at 0.31±0.07 and 0.61±0.08 mg/mL, respectively.

The reducing power of a compound may serve as a significant indicator of its potential antioxidant activity (34). A high absorbance value indicates strong reducing power. The reducing powers of the IPS, ISPS, SSe, and BHT were investigated. As shown in Fig. 5d, the reducing power of the ISPS increased with the increase of sample concentration. Compared with the IPS and SSe, ISPS showed higher reducing power. The reducing power of ISPS at 1.0 mg/mL was 0.45±0.01. Although the reducing capacity of ISPS was remarkably lower than that of BHT, ISPS still showed potential antioxidant activities.

### Changes in fasting blood glucose

Changes in fasting blood glucose are shown in Table 5. No significant differences among groups B, C, and D and groups B and C were found before treatment was administered. This result indicated that rats with diabetes were evenly grouped. The fasting blood glucose level of rats in group A at 2 weeks was not significantly different from that at 4 weeks after treatment was administered.

**Table 5:** Comparison of fasting blood glucose among the rats in the four groups

		After treatment	
		
Groups	Before treatment	2 weeks	4 weeks
A	5.49±0.54	5.21±0.37	5.19±0.26
B	15.82±0.73ΔΔ	21.63±0.81** ΔΔ ##	22.94±0.69** ΔΔ ##
C	15.79±0.84ΔΔ	15.67±0.55ΔΔ #	15.69±0.67ΔΔ
D	15.87±0.71ΔΔ	–	–

Compared with rats in group C

** *P*<0.01; compared with rats in group A

ΔΔ *P*<0.01; compared with the results before treatment

# *P*<0.05

## *P*<0.01.

### Changes in glycosylated serum protein

Changes in glycosylated serum protein are shown in Table 6. Glycosylated serum protein content of rats in group A was not significantly different from that of rats in group D at 2 weeks after treatment was administered (*P*>0.05); likewise, glycosylated serum protein content of rats in group B at 2 weeks was not significantly different from that at 4 weeks after treatment was administered (*P*>0.05). Glycosylated serum protein content of rats in group C at 2 weeks was not significantly different from that at 4 weeks after treatment was administered (*P*>0.05).

**Table 6:** Comparison between changes in glycosylated serum protein at 2 weeks and those at 4 weeks of rats in the four groups after treatment

Groups	2 weeks	4 weeks
A	2.09±0.12	2.07±0.13
B	4.25±0.31** ΔΔ	4.34±0.24** ΔΔ
C	3.12±0.21** ΔΔ	3.21±0.26** ΔΔ
D	2.11±0.14	2.13±0.12

Compared with rats in group A

** *P*<0.01; compared with rats in group D

ΔΔ *P*<0.01.

### Changes in malonaldehyde content

Changes in malonaldehyde content are shown in Table 7. At 2 weeks after treatment was administered, serum malonaldehyde content of rats in groups C and B was significantly higher than that of rats in group A (*P*<0.01). In contrast, serum malonaldehyde content of rats in group D was not significantly different from that in group A; likewise, serum malonaldehyde content of rats in group C was not significantly different from that of group B. At 4 weeks after treatment was administered, serum malonaldehyde content of rats in groups C and B was significantly higher than that of rats in groups A and D (*P*<0.01); serum malonaldehyde content of rats in group C was significantly lower than that in group B (*P*<0.01). Serum malonaldehyde content of rats in group B at 4 weeks was increased compared with that at 2 weeks (*P*<0.05), but this difference was not observed in group C. At 4 weeks, malonaldehyde contents in heart, pancreas, and kidney tissue in group B were significantly higher than those in groups A and D (*P*<0.01). However, changes in malonaldehyde content in various tissues of rats in group C were relatively complex. In particular, the malonaldehyde contents in heart, liver, and kidney tissues were significantly lower than that in group B (*P*<0.05); malonaldehyde in liver, pancreas, and kidney tissues was not significantly different from that of rats in group D; malonaldehyde in heart, liver, and pancreas tissues was higher than that of rats in group A. Malonaldehyde in liver tissue was significantly increased, but no significant difference in malonaldehyde in heart, pancreas, and kidney tissues was found in group D compared with that in group A.

**Table 7:** Malonaldehyde content in serum and tissue of rats in the four groups

		Serum (nmol/mL)		Tissue (nmol/mg prot)			
			
Groups	*n*	2 weeks	4 weeks	Heart	Liver	Pancreas	Kidney
A	6	5.04±0.62	5.31±0.41	0.52±0.07	0.63±0.08	0.41±0.02	1.13±0.13
B	8	10.58±0.87## ΔΔ	13.89±0.94## ΔΔ ▴	1.03±0.09## ΔΔ	1.09±0.05## ΔΔ	1.14±0.11## ΔΔ	2.09±0.14## ΔΔ
C	8	9.25±0.665##	9.37±0.77## ** ΔΔ	0.73±0.06** Δ	0.89±0.06## **	0.64±0.04#	1.44±0.12**
D	8	6.42±0.43	5.13±0.47	0.52±0.04	0.86±0.07##	0.47±0.04	1.15±0.11

Compared with rats in group A

# *P*<0.05

## *P*<0.01; compared with rats in group B

** *P*<0.01; compared with rats in group D

Δ *P*<0.05

ΔΔ *P*<0.01; compared with the results before 2 weeks

▴ *P*<0.05.

### Changes in total antioxidant capacity of rats with diabetes

Changes in total antioxidant capacity are shown in Table 8. At 4 weeks, serum total antioxidant capacity of rats in groups C, B, and D was significantly lower than that of rats in group A (*P*<0.01). Serum total antioxidant capacity of rats in groups C and B was significantly lower than that of rats in group D (*P*<0.01). Serum total antioxidant capacity of rats in group C was increased compared with that in group B (*P*<0.05). Total antioxidant capacity in heart, liver, pancreas, and kidney tissues of rats in group B was significantly lower than that of rats in groups A and D (*P*<0.01). Total antioxidant capacity in liver, pancreas, and kidney tissues of rats in group C was significantly increased compared with that of rats in group B (*P*<0.01). At 4 weeks, total antioxidant capacity in liver and kidney tissues of rats in group C remained significantly lower than that of rats in group A (*P*<0.01). Total antioxidant capacity in kidney tissue of rats in group C was significantly lower than that of rats in group D (*P*<0.01). Total antioxidant capacity in pancreas tissue of rats in group C was significantly lower than that of rats in group D (*P*<0.05). Total antioxidant capacity liver tissue of rats in group D remained significantly lower than that of rats in group A (*P*<0.01). Total antioxidant capacity in heart, pancreas, and kidney tissues of rats in group D was not significantly different from that of rats in group A.

**Table 8:** Total antioxidant capacity in serum and tissue of rats in the four groups

			Tissue (nmol/mg prot)			
			
Group	*n*	Serum (u/mL)	Heart	Liver	Pancreas	Kidney
A	6	8.72±0.78	0.51±0.04	1.29±0.10	0.81±0.07	1.10±0.06
B	8	3.38±0.13## ΔΔ	0.25±0.01## ΔΔ	0.47±0.03## ΔΔ	0.24±0.03## ΔΔ	0.34±0.02## ΔΔ
C	8	4.87±0.25## * ΔΔ	0.37±0.03	0.68±0.05## **	0.72±0.06** Δ	0.69±0.08## ** ΔΔ
D	8	6.92±0.54##	0.51±0.3	0.74±0.04##	0.97±0.08	1.02±0.13

Compared with rats in group A

## *P*<0.01; compared with rats in group B

* *P*<0.05

** *P*<0.01; compared with rats in group D

Δ *P*<0.05

ΔΔ *P*<0.01.

## Discussion

Streptozotocin is one of the most common reagents used to prepare diabetes experimental models; this reagent can selectively destroy pancreatic islet β cells to reduce insulin secretion and increase blood glucose of rats (35). At high glucose concentrations, glycosylated protein oxidation, aldehyde reduction in the cytoplasm, catecholamine secretion increase and oxidation promotion, and insufficient synthesis of the reduced form of nicotinamide–adenine dinucleotide phosphate together lead to an increase *in vivo* oxygen-free radicals; as a result, multiple oxidative damage occurs. Peroxidation of polyunsaturated fatty acids also occurs in the cell membrane, thereby forming lipid peroxide (such as malondialdehyde) (36). Serum total antioxidant capacity mainly refers to the sum of antioxidants in a non-enzymatic system and a few antioxidants of small molecular weight in an enzymatic system in the serum, which is one of the important indexes to determine body antioxidation function. At high glucose concentrations, total antioxidant capacity is significantly decreased (37). In this study, changes in malondialdehyde content and total antioxidant capacity in serum and tissues of rats in group B are consistent with these conclusions.

Glycosylated serum protein is the product of slow, consecutive non-enzymatic glycosylation reaction of serum protein at high blood glucose concentration. Glucose mainly combines with albumin of serum protein (38). The half-life of serum albumin is about 17 days; as such, glycosylated serum protein content can indicate the mean blood glucose level at 2 weeks–3 weeks before this parameter is measured. Therefore, glycosylated serum protein content is a reliable index to evaluate blood glucose control of patients at 2 weeks–3 weeks before sampling is conducted; a decrease in glycosylated serum protein can be detected at approximately 1 week after appropriate treatment is applied. It is not affected by hemoglobinopathy or other factors that can promote erythrocyte renewal; glycosylated serum protein can also be used to evaluate and monitor treatment efficacy for diabetes with high sensitivity and strong specificity (39). For instance, polysaccharides can reduce blood glucose level in several pathways, such as stimulating insulin synthesis and secretion, increasing insulin sensitivity of peripheral tissues, and regulating zinc metabolism (40). This study for the first time used glycosylated serum protein, along with changes in fasting blood glucose level, as an index to evaluate the hypoglycemic effect of ISPS at optimal concentrations. Either glycosylated serum protein or fasting blood glucose level indicated that ISPS significantly reduced blood glucose level but could not control blood glucose level in an ideal manner. Glycosylated serum protein and fasting blood glucose levels at 4 weeks after treatment were not further decreased as compared to those at 2 weeks. Fasting blood glucose level of rats in group B at 4 weeks after treatment was significantly increased compared with that at 2 weeks, but glycosylated serum protein was not statistically different possibly because the increase in blood glucose level did not change the glycosylated serum protein content. Likewise, fasting blood glucose and glycosylated serum protein at 4 weeks after treatment in group C were similar to those at 2 weeks; nevertheless, this result should be further investigated to determine whether this increase occurred because ISPS can maintain the blood glucose level of rats with diabetes at a certain range or because changes in blood glucose level of rats in that group are not significant.

The effect of ISPS on malondialdehyde and total antioxidant capacity of rats with diabetes (*in vivo*) was also investigated in the study. ISPS could significantly reduce serum malondialdehyde content and increase total antioxidant capacity at 4 weeks after treatment was administered. Considering hyperglycemic factor, we designed group D with strictly controlled blood glucose level. We found that malondialdehyde content and total antioxidant capacity in most tissues of rats in group D were recovered to normal levels, but significant abnormalities were observed in individual tissues.

## Conclusions

Thin stillage was successfully utilized as the substrate of *C. sinensis* to produce ISPS. Strains grew well, and a high yield of ISPS was obtained. Production of ISPS was optimized by adding SSe. In addition, the statistical analysis proved the usefulness and efficiency of the proposed method in the optimization of fermentation conditions, providing a valuable reference for both laboratory and commercial production of ISPS. The ISPS exhibited a strong antioxidative ability *in vitro*, indicating that the ISPS of *C. sinensis* can be used as a potential antioxidant to enhance adaptive immune responses. In addition, ISPS can reduce blood glucose level and improve antioxidant capacity of rats with diabetes induced by streptozotocin.
